# OSbrca: A Web Server for Breast Cancer Prognostic Biomarker Investigation With Massive Data From Tens of Cohorts

**DOI:** 10.3389/fonc.2019.01349

**Published:** 2019-12-20

**Authors:** Zhongyi Yan, Qiang Wang, Xiaoxiao Sun, Bingbing Ban, Zhendong Lu, Yifang Dang, Longxiang Xie, Lu Zhang, Yongqiang Li, Wan Zhu, Xiangqian Guo

**Affiliations:** ^1^Cell Signal Transduction Laboratory, Department of Preventive Medicine, Bioinformatics Center, School of Basic Medical Sciences, School of Software, Institute of Biomedical Informatics, Henan University, Kaifeng, China; ^2^Department of Anesthesia, Stanford University, Stanford, CA, United States

**Keywords:** survival, breast cancer, prognosis, biomarker, OSbrca

## Abstract

Potential prognostic mRNA biomarkers are exploited to assist in the clinical management and treatment of breast cancer, which is the first life-threatening tumor in women worldwide. However, it is technically challenging for untrained researchers to process high dimensional profiling data to screen and validate the potential prognostic values of genes of interests in multiple cohorts. Our aim is to develop an easy-to-use web server to facilitate the screening, developing, and evaluating of prognostic biomarkers in breast cancers. Herein, we collected more than 7,400 cases of breast cancer with gene expression profiles and clinical follow-up information from The Cancer Genome Atlas and Gene Expression Omnibus data, and built an Online consensus Survival analysis web server for Breast Cancers, abbreviated OSbrca, to generate the Kaplan–Meier survival plot with a hazard ratio and log rank *P*-value for given genes in an interactive way. To examine the performance of OSbrca, the prognostic potency of 128 previously published biomarkers of breast cancer was reassessed in OSbrca. In conclusion, it is highly valuable for biologists and clinicians to perform the preliminary assessment and validation of novel or putative prognostic biomarkers for breast cancers. OSbrca could be accessed at http://bioinfo.henu.edu.cn/BRCA/BRCAList.jsp.

## Introduction

Breast cancer is one of the leading cancers and the primary cause of mortality in women. The global burden of breast cancer is still increasing (1). It is predicted that by 2021, the incidence of breast cancer will increase to 85 per 100,000 women in China (2). Currently, clinicopathological risk factors are primarily used to estimate prognosis. These clinicopathological risks include stage, histological grade, tumor size, lymph node infiltrate, and so on (3). Molecular subtypes influence the survival of breast cancer. According to three protein expression statuses [estrogen receptor, progesterone receptor, and human epidermal growth factor receptor 2 (HER2)], breast cancer can be categorized into four classes: luminal A, luminal B, basal-like, and HER2+ (4). Because of the heterogeneity and survival difference of breast cancer, the utmost interests for researchers are how to validate the prognostic and predictive candidate genes in appropriately powered breast cancer cohorts using the massive published expression levels of various genes profiles with clinical outcome.

So far, a number of poor clinical outcome associated genes have been identified. The most famous prognostic significance of breast cancer is the estrogen receptor gene, which is expressed in 50–70% of clinical tumor cases (5). Progesterone receptor and HER2 are two other important prognostic-related and predictive genes for breast cancer. In addition, a lot of new prognostic genes are exploited for diagnosing and curing breast cancer, such as breast cancer 1/2, TP53, cyclin D1, cyclin E, cathepsin D, cystatin E/M, and plexin B1 (6–8). Many studies showed that using multigenes as a panel of biomarkers may work more accurately to predict clinical outcome (9). Therefore, multivariate cohorts are needed to identify novel genes, and these genes need to be exploited to cure and evaluate prognosis of breast cancer.

By combining clinical follow-up data and high-throughput profiling data, we have reached a better understanding in the study of breast carcinoma. In this study, we collected the gene expression profiling data with follow-up information of breast cancers, which were mainly from The Cancer Genome Atlas (TCGA) and Gene Expression Omnibus (GEO) database. Our aim is to provide a high powerful web server with massive data to generate survival plots to assess the relevance of the expression levels of interested genes on the clinical outcome for breast cancer patients. The Online consensus Survival analysis web server for Breast Cancers offers a web server to clinicians or non-bioinformatics researchers to appraise or exploit potential prognostic genes. Users can predict the prognostic potency of gene of interests using OSbrca.

## Methods and Experiment

### Data Collection

The gene expression profiling datasets for breast cancer were mainly composed of TCGA and GEO cohorts (Table 1) according to the following four criteria: (1) the cohort must have at least 50 breast cancer cases, (2) the cohort must contain individual clinical follow-up information, (3) the probe annotation should be completed or probe could be translated to gene symbol by ID conversion, such as DIVID, and (4) only platforms with more than 50 individual samples were selected if GEO cohorts having more than one platform.

**Table 1:** The basic information of The Cancer Genome Atlas (TCGA) and Gene Expression Omnibus (GEO) of breast cancer cohorts in Online consensus Survival analysis web server for Breast Cancers (OSbrca).

	**Datasets**	**Cohort**	**Platform**	**Survival**	**No.^#^**	**References**
1	NIH and NHGRI	TCGA	DCC	OS, PFI, PFS, DSS, DFI	1,083, 1,096, 1,096, 1,078,952	(19)
2	Chapel Hill	GSE10885	GPL1390	OS, RFS	94, 95	(20)
3	Chapel Hill	GSE10886	GPL1390	OS, RFS	178, 178	(21)
4	Chapel Hill	GSE10893	GPL1390	OS, RFS	155, 156	(22)
5	Leverkusen	GSE11121	GPL96	MFS	200	(23)
6	San Diego	GSE12093	GPL96	DFS	136	(24)
7	Rotterdam	GSE12276	GPL570	MFS	204	(25)
8	Carlsbad	GSE1379	GPL1223	DFS	60	(26)
9	Stockholm	GSE1456	GPL96	OS, MFS	159, 159	(27)
10	Woburn	GSE17705	GPL570	RFS	298	(28)
11	Chapel Hill	GSE18229	GPL887	OS, RFS	53, 53	(29)
12	Chapel Hill	GSE18229	GPL1390	OS, RFS	164, 165	(29)
13	San Diego	GSE2034	GPL96	MFS	286	(30)
14	Taipei	GSE20685	GPL570	OS, MFS	327, 327	(31)
15	Toronto	GSE20711	GPL570	OS, RFS	88, 88	(32)
16	Marseille	GSE21653	GPL570	DFS	248	(33, 34)
17	Helsinki	GSE24450	GPL6947	OS, DFS	183, 183	(35, 36)
18	New York	GSE2603	GPL96	MFS	82	(37)
19	Chapel Hill	GSE2607	GPL1390	OS, RFS	52, 52	(38)
20	Chapel Hill	GSE26338	GPL887	OS, RFS	56, 56	(39)
21	Chapel Hill	GSE26338	GPL1390	OS, RFS	173, 174	(39)
22	Köln	GSE26971	GPL96	MFS	258	(40)
23	Chapel Hill	GSE2741	GPL1390	OS, RFS	61, 61	(41)
24	Toronto	GSE2990	GPL96	RFS	109	(42)
25	Amsterdam	GSE31364	GPL14378	DFS	72	(43)
26	Durham	GSE3143	GPL8300	OS	158	(44)
27	Marseille	GSE31448	GPL570	DFS	251	(34)
28	Taipei	GSE33926	GPL7264	MFS	51	(45)
29	Singapore	GSE3494	GPL96	DSS	237	(46)
30	Chapel Hill	GSE3521	GPL1390	OS, RFS	84, 84	(47)
31	Chapel Hill	GSE35629	GPL1390	OS, RFS	53, 53	(48)
32	Milan	GSE37181	GPL6884	MFS	123	(49)
33	Bethesda	GSE37751	GPL6244	OS	61	(50)
34	Bethesda	GSE39004	GPL6244	OS	61	(50, 51)
35	Bangalore	GSE40206	GPL4133	MFS	61	(52)
36	Amsterdam	GSE41994	GPL16233	DFS	103	(53)
37	Dublin	GSE42568	GPL570	OS, RFS	104, 104	(54)
38	Winston-Salem	GSE45255	GPL96	DFS, MFS, DSS	94, 136, 134	(55)
39	Taipei	GSE48391	GPL570	DFS	81	(56)
40	Singapore	GSE4922	GPL96	DFS	249	(57)
41	Chicago	GSE5327	GPL96	MFS	58	(58)
42	Taipei	GSE53752	GPL7264	MFS	51	(45)
43	Chapel Hill	GSE6130	GPL1390	OS, RFS	86, 87	(59)
44	Toronto	GSE6532	GPL96	RFS, MFS	119, 239	(60–62)
45	Toronto	GSE7390	GPL96	OS, RFS, MFS	198, 198, 198	(63, 64)
46	Toronto	GSE9195	GPL570	RFS, MFS	77, 77	(61, 62)
47	Montpellier	GSE9893	GPL5049	OS, DFS	155, 155	(65)
Total^##^					7456	

# The number of samples only includes follow-up information;

## *only the sum of the highest survival number. OS, overall survival; PFI, progression-free interval; PFS, progression-free survival; DSS, disease-specific survival; DFI, disease-free interval; RFS, recurrence-free survival; DFS, disease-free survival; MFS, metastasis-free survival*.

### Development of OSbrca

The OSbrca server is deployed in a tomcat server as previously described with minor modification (10). In brief, front-end application was exploited in HTML and JSP to retrieve user inputs and display the output on the web page. Java and R were also used in the server application to control the analysis request and return the results. The gene expression profiles and clinical data were stored and managed by the SQL Server database. The R and SQL Server were linked by third middleware (The R packages, “RODBC” and “JDBC”). The R package “survminer” and “survival” generate Kaplan–Meier (KM) survival curves with log-rank *P*-value and calculate the hazard ratio (HR) with 95% confidence intervals (95%CI). The KM survival curves measure the effect of genes on survival using breast cancer data (11). Log-rank test is the standard method of survival data comparison, which is widely used in survival analysis (12). HR and 95% confidence interval (95% CI) were calculated by univariate Cox regression analysis. OSbrca can be accessed in http://bioinfo.henu.edu.cn/BRCA/BRCAList.jsp.

### Collection and Authenticating Previously Reported Prognostic Biomarkers of Breast Cancer

To collect previously published biomarkers of breast cancer in the PubMed, three key words were used: breast cancer, prognostic, and biomarker. One hundred and twenty-eight previously identified prognostic biomarkers are listed in Table S1. To examine the performance of OSbrca, each reported prognostic biomarker was analyzed in OSbrca, by categorizing patients with “upper 25%” (the upper 25% expression vs. the bottom 75% expression). In addition, OSbrca is a web server for cross-validation of the potential prognostic biomarkers among tens of breast cancer cohorts. As a result, the methodology of validation in OSbrca includes two parts. First, we performed the validation of prognostic biomarkers between different breast cancer cohorts, and this independent validation between cohorts is of great importance for biomarker development; second, validation of previously reported prognostic biomarkers in OSbrca presented the reliability of OSbrca.

## Results

### Collection of Gene Expression Profiles With Clinical Follow-Up Information of Breast Cancer

Breast cancer is the leading mortality in women and is one of the most widely studied cancers. Thus, the urge for breast cancer patient is to exploit novel therapy target and prognostic biomarkers, which would offer the opportunities to assist the clinical management and treatment. However, it is technically challenging for untrained researchers to process the high dimensional profiling data to screen and validate the potential prognostic values of genes of interests in multiple cohorts. To build OSbrca, we have collected more than 7,400 samples of breast cancer expression profiles with clinical follow-up information, mainly obtained from TCGA (1,092 samples) and GEO cohorts (6,364 samples) (Table 1). OSbrca includes overall survival (OS, 3,786 patients from 23 cohorts), progression-free interval (1,096 patients only from TCGA cohort), progression-free survival (1,096 patients only from TCGA cohort), disease-specific survival (1,499 patients from three cohorts), disease-free interval (952 patients only from TCGA cohort), recurrence-free survival (RFS, 2,207 patients from 19 cohorts), disease-free survival (DFS, 1,632 patients from 11 cohorts), and metastasis-free survival (MFS, 2,508 patients from 16 cohorts). In other words, the OSbrca can predict those eight survival endpoints basing on breast cancer clinical information, such as RFS.

### The Architecture of the OSbrca Web Server for Breast Cancer

Based on the expression profiles and clinical outcome of breast cancers, OSbrca can determine the prognostic values of interested genes using KMPlot, HR, and log-rank *P*-value. OSbrca has implemented several optional clinical confounding factors, such as data source, age, stage, histological type, molecular subtype, survival, and ER/PgR/HER2 status. Users can select different cutoff, such as the upper 25%, for gene expression levels when categorizing the breast cancer population. The interface of the OSbrca is simple and friendly. Users could input the particular official gene symbol with all the default parameters and then click “Kaplan–Meier plot” button. The KMPlot with HR and log-rank *P*-value will be displayed on the output web page.

### Evaluation of the Previously Reported Prognostic Biomarkers of Breast Cancer in OSbrca

**Figure 1:**
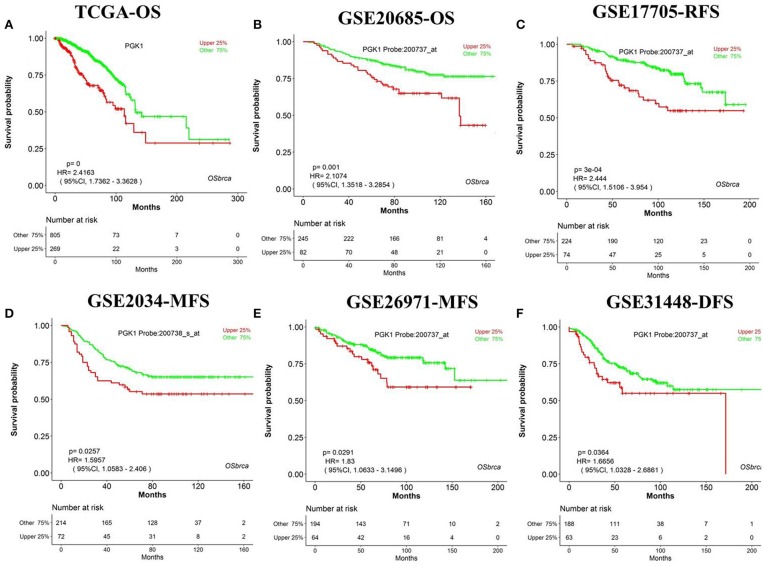
Validation previously reported gene *PGK1* in Online consensus Survival analysis web server for Breast Cancers (OSbrca). *PGK1* gene is high expressed in tumor tissue as a worse prognostic survival biomarker in breast cancer. **(A)** OS of TCGA, **(B)** OS of GSE20685, **(C)** RFS of GSE17705, **(D)** MFS of GSE2034, **(E)** MFS of GSE269721, **(F)** DFS of GSE31448. *PGK1*, phosphoglycerate kinase 1; OS, overall survival; DFS, disease-free survival; RFS, recurrence-free survival; MFS, metastasis-free survival.

**Figure 2:**
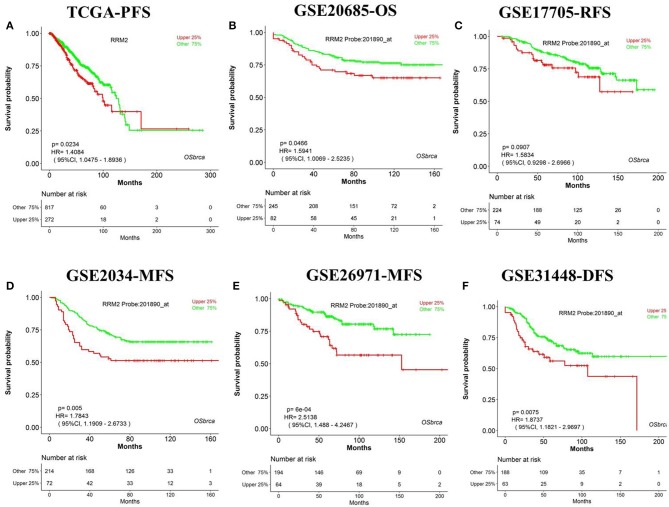
Validation previously reported gene *RRM2* in Online consensus Survival analysis web server for Breast Cancers (OSbrca). *RRM2* gene is a poor prognostic biomarker in breast cancer. **(A)** Progression-free survival (PFS) of The Cancer Genome Atlas (TCGA); **(B)** OS of GSE20685; **(C)** RFS of GSE17705; **(D)** MFS of GSE2034; **(E)** MFS of GSE269721; **(F)** DFS of GSE31448. *RRM2*, ribonucleotide reductase regulatory subunit M2; PFS, progression-free survival; OS, overall survival; RFS, recurrence-free survival; MFS, metastasis-free survival; DFS, disease-free survival.

We have designed OSbrca to be a user-friendly and easy-to-use online web server to analyze and evaluate the prognostic values of particular genes in 48 breast cancer cohorts using existing high-throughput profiling breast cancer data. To measure the performance and determine the reliability of OSbrca, we have collected previously published prognostic biomarkers of breast cancer (Table S1) and tested their prognostic potency in OSbrca. Fu et al. have demonstrated that PGK1 was overexpressed in tumor tissue and was an indication of worse survival biomarker in breast cancer (13). Using OSbrca, we showed that *PGK1* gene was indeed a poor survival biomarker in breast cancer cohorts (top 6 samples): TCGA [OS, HR (95% CI) = 2.42 (1.74–3.36), *P* < 0.0001], GSE20685 [OS, HR (95% CI) = 2.11 (1.35–3.39), *P* = 0.001], GSE17705 [RFS, HR (95% CI) = 2.44 (1.51–3.95), *P* < 0.001], GSE2034 [MFS, HR (95% CI) = 1.60 (1.06–2.41), *P* = 0.0257], GSE269721 [MFS, HR (95% CI) = 1.83 (1.06–3.15), *P* = 0.0291], and GSE31448 [DFS, HR (95% CI) = 1.67 (1.03–2.69), *P* = 0.0364] (Figure 1). We also test another reported poor DFS biomarker RRM2. Figure 2 shows that *RRM2* gene was an indication of worse survival indicator in five out of six breast cancer cohorts (top 6 samples), except in the cohort of GSE17705 (Figure 2). One hundred and twenty-eight previous reported prognostic biomarkers were validated in OSbrca shown in Table S1. Based on our studies using OSbrca, 62% analyzed biomarkers (79/128) showed consistent performance as reported in the literature, but some biomarkers showed contradictory outcomes to previous results. Taking the *AOCA1* gene as an another example, a previous study showed that the *AOCA1* gene could potentially predict a worse clinical prognosis in breast cancer (14). However, the analysis from OSbrca suggested that breast cancer patients with the overexpression of the *AOCA1* gene would potentially have a better clinical outcome (Table S1). In summary, all the results showed that the OSbrca web server is very reliable through validating previously reported biomarkers of breast cancer.

## Discussion

Breast cancer is widely profiled by RNA-sequences and gene microarrays, such as TCGA. Thus, the core and focus issue is how to excavate potential therapy targets and to develop prognostic biomarkers by possessing massive high-throughput profiles. Based on massive data of different cohorts, we integrated 48 cohorts of breast cancer datasets and established an online web server, named OSbrca. OSbrca implanted a selective set of clinical parameters, including tumor grade, age, status of ER/PgR/HER2, menopause status, and so on. The OSbrca could output the KMPlot with HR and log rank *P*-value for given genes in an interactive way. In addition, users can study genes in a particular country or race using OSbrca, such as Chinese breast cancer patients. Herein, we retrospectively validated the previously reported prognostic biomarkers of breast cancer. The results showed that most previous reported biomarkers could be identified by some different cohorts of OSbrca (Figures 1, 2, and Table S1). In addition, OSbrca is an across-validation web server used to exploit breast cancer biomarkers based on different independent cohorts of breast cancer. Cross-validation in OSbrca means that it is important to exploit prognostic biomarkers among tens of breast cancer cohorts and also presents the reliability of OSbrca.

So far, there are some online prognostic websites for breast cancer, such as KM plotter (11), PROGgene (15), ITTACA (16), PrognoScan (17), OncoLnc, and GEPIA (18), but the size of datasets used in these tools is relatively small and limited compared to OSbrca. Specifically, OSbrca integrates 48 cohorts that contain more than 7,400 patients with RNA-sequencing and gene microarray data. It allows researchers to revisit previous protein biomarkers and exploit novel prognostic biomarkers. There are some limitations of this study, such as the loss of different platform integration, lacking noncoding gene information, which will be solved in the new-version of this tool. In addition, when new cohorts become available, we will update OSbrca in a timely manner.

In conclusion, the OSbrca web server integrates more than 7,400 follow-up breast samples and is highly valuable for researchers with a limited bioinformatics background to access and uncover prognostic-related biomarkers for breast cancer.

## Data Availability Statement

The data for this manuscript can be accessed at OSbrca http://bioinfo.henu.edu.cn/BRCA/BRCAList.jsp. The raw data supporting the conclusions of this manuscript will be made available by the authors, without undue reservation, to any qualified researcher.

## Author Contributions

XG: research design. ZY, XS, LX, BB, ZL, YD, WZ, and LZ: collect and deal with data. ZY, LX, XS, YD, LZ, YL, and XG: draft of the manuscript. QW and XG: establish OSbrca Web Server. BB, XS, ZL, and YL: collect and validate previous reported biomarkers of breast cancer. ZY, QW, XS, LX, BB, YD, LZ, WZ, and XG: critical revision of the manuscript.

### Conflict of Interest

The authors declare that the research was conducted in the absence of any commercial or financial relationships that could be construed as a potential conflict of interest.
